# Prognosis is still poor in patients with posttransplant C3 glomerulopathy despite eculizumab use

**DOI:** 10.1093/ckj/sfae190

**Published:** 2024-06-20

**Authors:** Safak Mirioglu, Rabia Hacer Hocaoglu, Arzu Velioglu, Yasemin Ozluk, Ahmet Burak Dirim, Aysegul Oruc, Ozgur Akin Oto, Halil Yazici, Yasar Caliskan

**Affiliations:** Division of Nephrology, Istanbul University Istanbul Faculty of Medicine, Istanbul, Turkey; Department of Immunology, Istanbul University Aziz Sancar Institute of Experimental Medicine, Istanbul, Turkey; Division of Nephrology, Istanbul University Istanbul Faculty of Medicine, Istanbul, Turkey; Division of Nephrology, Marmara University School of Medicine, Istanbul, Turkey; Department of Pathology, Istanbul University Istanbul Faculty of Medicine, Istanbul, Turkey; Division of Nephrology, Istanbul University Istanbul Faculty of Medicine, Istanbul, Turkey; Division of Nephrology, Bursa Uludag University Faculty of Medicine, Bursa, Turkey; Division of Nephrology, Istanbul University Istanbul Faculty of Medicine, Istanbul, Turkey; Division of Nephrology, Istanbul University Istanbul Faculty of Medicine, Istanbul, Turkey; Division of Nephrology, Istanbul University Istanbul Faculty of Medicine, Istanbul, Turkey; Center for Abdominal Transplantation, Saint Louis University School of Medicine, St Louis, MO, USA

To the Editor,

C3 glomerulopathy (C3G) is known for its tendency to recur after kidney transplantation (KTx), and recurrence has been reported to be as high as 86% in contemporary cohorts with a high rate of graft failure [1]. The usual maintenance immunosuppressive regimen after KTx includes a calcineurin inhibitor, a mycophenolic acid (MPA) derivative and a corticosteroid which may have some therapeutic effect on the recurrent disease itself, and eculizumab can be used as an additive treatment option in patients with high-grade proteinuria and/or progressive estimated glomerular filtration rate (eGFR) loss [2]. However, data from various cohorts are still needed, especially regarding treatment responses and outcomes of patients.

In this retrospective multicenter study conducted across three centers, we collected the data of kidney transplant recipients (KTRs) who were diagnosed with posttransplant recurrent or *de novo* C3G between 2014 and 2023 and followed for at least 3 months after diagnosis. Demographic, clinical, laboratory and histopathological characteristics of patients were retrieved from the databases of participating centers. Primary outcome was defined as death-censored graft loss necessitating dialysis or re-transplantation, and secondary outcome was complete (CR) or partial remission (PR). CR was the recovery of baseline eGFR and proteinuria of <0.5 g/g. PR was ≥50% reduction of proteinuria (and to <3 g/g in patients with nephrotic-range proteinuria at baseline) plus stabilization or improvement in kidney function. Included patients provided informed consent to extract their data to the database. Use of this database for research was approved by the Istanbul University Istanbul Faculty of Medicine Ethical Committee (2016/742), and the study complied with the Declaration of Helsinki and its later amendments.

Eleven patients were identified, and 10 with follow-up data were included. Detailed features of patients are shown in the Table 1. Five (50%) were male, and mean (± standard deviation) age at the time of KTx was 33.2 ± 8.5 years. Nine KTRs (90%) were diagnosed with recurrent C3G and the etiology of primary kidney disease was not known in one patient. The majority of KTx were performed from living donors (*n* = 9, 90%). One patient had a history of T-cell-mediated rejection before posttransplant C3G diagnosis, which had shown good response to anti-rejection treatment. Posttransplant C3G was diagnosed at a median of 26 (3–85) months after KTx, and mean age was 36.8 ± 9.1 years. Mean hemoglobin, serum creatinine, serum albumin and proteinuria at the time of diagnosis were 10.5 ± 1.8 g/dL, 1.9 ± 0.7 mg/dL, 4.1 ± 0.5 g/dL and 1.1 ± 0.9 g/g, respectively. Monoclonal disorders were excluded by using serum and urine electrophoresis and serum free light chain assays in all KTRs. Serum C3 was low (67.1 ± 29.7 mg/dL, ref: 90–180 mg/dL) in seven of eight KTRs with available data (87.5%). At the time of diagnosis, eight patients were treated with tacrolimus, MPA and low-dose prednisolone, and the combination of tacrolimus, azathioprine (AZA) and low-dose prednisolone was administered in two patients. Antiproliferative agent was changed from AZA to MPA after diagnosis in one patient. Further immunosuppressive treatment was administered in nine cases for a median of 14.5 (3–24.3) months. Eculizumab was used in eight, and one patient was treated with pulse steroids and therapeutic plasma exchange. Four of eight patients (50%) who were treated with eculizumab showed CR. One patient treated with steroids and therapeutic plasma exchange did not go into remission. Response to treatment was seen in these patients after 3 (1.5–6.8) months (1, 8, 3 and 3 months for patients #1, #2, #8 and #10, respectively). After a median of 27 (12.3–74.5) months, five KTRs (50%) experienced graft loss despite eculizumab use in three of them. Patient #4 progressed to graft failure after 4 months, and patient #6 underwent re-transplantation due to graft failure in 8 months after recurrence. Patients #5 and #7 demonstrated a significant increase in serum creatinine levels at 1 year which resulted in graft loss. No adverse events attributed to treatment were observed in the whole cohort.

**Table 1:** Detailed characteristics of all patients with posttransplant C3 glomerulopathy (reference for serum C3 levels: 90–180 mg/dL).

Patient	Sex	Primary disease	Family history	Age at KTx	Donor type	Post-KTx Diagnosis	Age at post-KTx diagnosis	Time from KTx to diagnosis (months)	Biopsy indication	Endocapillary proliferation	Percentage of cellular and/or fibrocellular crescents	Interstitial inflammation	Percentage of sclerotic glomeruli	Interstitial fibrosis	Tubular atrophy	SCr at diagnosis (mg/dL)	Serum C3 at diagnosis (mg/dL)	SCr 1 year after diagnosis (mg/dL)	Time from diagnosis to last follow-up (months)	Treatment	Remission	Graft loss
1	F	C3G	None	38	Living	C3G	40	24	Graft dysfunction and hematuria	No	0	Yes	16.7	Mild	Mild	2.10	81	1.30	33	ECZ	CR	No
2	F	C3G	None	46	Living	C3G	49	28	Proteinuria and hematuria	No	14.3	Yes	0	None	None	1.60	89	1.36	32	ECZ	CR	No
3	F	C3G	KTx in aunt	21	Living	DDD	21	7	Proteinuria and hematuria	No	18.2	Yes	9.1	Mild	Mild	1.35	15	0.97	15	ECZ	NR	No
4	M	Unknown	None	36	Living	C3G	45	109	Graft dysfunction, proteinuria and hematuria	No	10	Yes	25	Mild	Mild	3.00	56	Graft loss	4	ECZ	NR	Yes
5	M	MPGN	None	25	Living	C3G	29	48	Proteinuria and hematuria	No	0	Yes	40	None	Mild	1.39	81	3.28	16	ECZ	NR	Yes
6	M	MPGN	None	25	Living	C3G	36	139	Graft dysfunction	No	0	No	20	Mild	Moderate	3.50	NA	Graft loss and re-KTx	97	None	NA	Yes
7	F	C3G	None	36	Deceased	C3GN	43	77	Graft dysfunction and proteinuria	Yes	3.2	Yes	12.9	Mild	Mild	1.80	NA	2.50	67	ECZ	NR	Yes
8	M	C3G	None	29	Living	C3G	29	1	Graft dysfunction	Yes	0	No	0	None	Mild	1.42	40	1.02	22	TPE, then ECZ	CR	No
9	M	MPGN	KTx in father	31	Living	C3G	31	3	Proteinuria	No	0	No	0	None	Mild	1.33	109	1.36	121	TPE with steroids	NR	Yes
10	F	C3G	None	45	Living	C3G	45	3	Graft dysfunction	No	0	Yes	0	None	None	1.90	66	NA	3	Steroids and ECZ	CR	No

C3GN: C3 glomerulonephritis; DDD: dense deposit disease; ECZ: eculizumab; F: female; M: male; MPGN: membranoproliferative glomerulonephritis; NA: not available; NR: no remission; SCr: serum creatinine; TPE: therapeutic plasma exchange.

Prognosis was still quite dismal in our cohort despite eculizumab use in the majority of the patients. Our results were in line with the findings of the contemporary cohorts [1, 3, 4]. However, our observations suffer from limitations. This is a retrospective study which precludes any cause–effect relationship, and we do not have information on antibodies for complement proteins or genetic analysis. We could not perform a centralized pathology review for biopsy samples, and reported already available biopsy data which precluded providing further detailed information on all histologic activity parameters [5]. Dichotomization of C3G into C3 glomerulonephritis and dense deposit disease subtypes was not possible in most patients due to the lack of electron microscopy. Eculizumab was not found to be very beneficial in patients with a quiescent progressive course instead of a crescentic rapidly progressive disease in native kidneys [6], which might also be the case in posttransplant disease. Studies on new potentially effective treatment options such as novel inhibitors of the alternative pathway, like iptacopan [7], are urgently needed.
